# Continuous overnight monitoring of body temperature during embryo transfer cycles as a proxy for establishing progesterone fluctuations by comparison with P4 blood progesterone results: a prospective, observational study

**DOI:** 10.1186/s12958-024-01329-0

**Published:** 2025-02-03

**Authors:** T.G. Knowles, J.A. García-Velasco, M. Toribio, N. Garrido, A.I. Barrio Pedraza, C. Colomé Rakosnik, A. Salazar Vera, R. Milnes

**Affiliations:** 1 Faculty of Health Sciences, University of Bristol, Bristol, UK; 2 IVIRM Global Research Alliance, IVIRMA Madrid, Av. del Talgo, 68, Moncloa - Aravaca, Madrid 28023 Spain; 3 IVIRM Global Research Alliance Fundación IVI, Hospital Universitario y Politécnico La Fe, Avenida Fernando Abril Martorell, 106 - Biopolo, Torre A, Planta 1ª, Valencia 46026 Spain; 4 IVIRM Global Research Alliance IVIRMA Mallorca, C/ Antoni Villalonga 6, Palma de Mallorca, 07011 Spain; 5 IVIRM Global Research Alliance IVIRMA Málaga, Avenida de la Aurora 55, Málaga 29006 Spain; 6 viO HealthTech Limited, Unit 211, Basepoint Business Centre, 377–399 London Road, Camberley, GU15 3HL UK

## Abstract

**Background:**

A prospective, observational study to identify relationships between body temperature and levels of peripheral P4 blood progesterone, and examine if these differ according to body temperature cycle pattern.

**Methods:**

62 data points from 18 patients undergoing hormone assisted embryo transfer cycles at IVIRMA IVF clinics in Madrid, Mallorca and Malaga, Spain volunteered to use OvuSense, an intra-vaginal body temperature monitor. Primary outcome measures were OvuSense Raw and Smooth Temperature (ST) (°C), P4 (ng/ml). Secondary outcome measures: Ongoing Pregnancy, Miscarriage or biochemical pregnancy. Graphical time based comparison analyses and multilevel regression analyses using MLwiN 3.10 [Charlton C, Rasbash J, Browne WJ, Healy M, Cameron B. MlwiN Version 3.10. Centre for Multilevel Modelling. University of Bristol; 2024.] software were conducted.

**Results:**

A graphical analysis showed an apparent relationship between P4 levels and Temperature taken on P4 blood draw day. A multilevel regression analysis using MLwiN 3.10 Centre for Multilevel Modelling. University of Bristol software investigated this relationship, allowing between-patient variation to be accounted for and estimated. This established a strong linear relationship between LnP4 and ST, and cross correlation was carried out which identified the optimum predictor of levels of LnP4 was ST measured on the day prior to blood sampling. Further graphical analyses showed an apparent lower luteal level of P4 for cycles flagged as atypical by OvuSense, and for negative outcomes, except on embryo transfer day.

**Conclusions:**

The results provide extremely strong evidence of a linear relationship between LnP4 and Smooth Temperature (ST) measured the day before blood sampling (Z = 15.6, *p* < 0.0001, 2 sided). This suggests that ST could provide a less invasive, continuous, and more practical method of assessing P4 response. Secondary outcomes may be related to ST pattern established during an embryo transfer cycle. Further investigation is required to establish the value of the ST pattern for improving outcomes.

## Introduction

Barton and Wiesner (1945) [1] confirmed the bi-phasic temperature pattern established by van de Velde (1904) [2]. They established the rise associated with ovulation to be around 0.6 degrees Fahrenheit (0.32 °C), postulating that temperature rises due to the thermogenic effect of released progesterone. Temperature charts that measure basal body temperature (BBT) upon waking have therefore been used for many years to confirm ovulation.

Traditional clinical thinking is that progesterone starts being released after a follicle has ruptured and the corpus luteum starts to form and therefore a temperature rise can only take place after ovulation has occurred. Recent research questions this thinking, suggesting that there is a progesterone rise of approximately 0.5 ng/ml prior to ovulation [3].

The OvuCore sensor is worn vaginally, to predict and confirm ovulation. It measures core body temperature every 5 min overnight, rather than using a simple, single measure as in BBT. The findings of initial clinical trials for OvuSense conducted in 2012 [4–7], confirmed the ideas originally put forward by Barrett and Marshall (1969) [8], which suggest that ovulation has occurred if in any window of 6 consecutive days there is a sustained rise of over 0.1 degrees Celsius for each day of 3 consecutive days. This work was later supported by McCarthy and Rockette (1983) [9] and forms the basis of the proprietary OvuSense algorithm. The ability of core body temperature to more accurately track the level of progesterone and to provide real time prediction of ovulation is confirmed by Coyne al. (2000) [10].

Clinical literature examining the use of BBT or other temperature methods in IVF is scarce. Forman et al. (1987), studied the effect of endogenous progesterone on BBT in stimulated ovarian cycles concluding that, in the first four days of the luteal phase, there was no correlation between rise in progesterone and differential increase in BBT [11]. Mordel et al. (1992) reached a different conclusion in the case of cycles with an hCG trigger shot, noting that there was a significant correlation (p < 0.0002) between rise in BBT and increase in progesterone after hCG administration” [12].

The main study objective was to identify if any relationships exist between measurements of body temperature taken with the OvuSense device, and levels of P4 measured at up to five time points during the cycle. The secondary objective was to determine if progesterone patterns throughout the cycle, as determined by continuous temperature measurements using OvuSense correlate with embryo transfer outcomes.

## Methods

### Sample size

A convenience sample of 50 was adopted based upon the rate at which it was anticipated women could be recruited to the study over a reasonable timespan of one year.

## Intermediate analysis

An intermediate analysis was proposed once complete cycle data from 18 women became available, to determine whether to continue or cease further data collection. If an exploratory analysis failed to find a relationship at α ≤ 0.2, data collection would cease. The analysis was performed by Knowles T. in consultation with Milnes R., García-Velasco JA. and Garrido N, and subsequently reviewed with the remaining authors in the context of this paper.

A regression analysis indicated a highly significant linear relationship between ST and LnP4 (Z = 14.738, *p* < 0.0001), and further analyses confirmed sufficient merit in the data in the opinion of the authors to warrant publication.

However, recruitment for the study was found to be somewhat impaired in two of the clinics due to the increasing adoption of ‘natural cycle IVF’ (without hormone replacement therapy), and the authors in turn questioned whether natural IVF cycles would show different relationship between ST and LnP4. It was therefore decided to continue the study with a modified protocol including these patient types. The remainder of this paper shall focus on the intermediate analysis with the 18 patients.

### Study design

This is a study of women undergoing IVF treatment who volunteer to measure body temperature with the OvuCore intra-vaginal sensor overnight and download this to the OvuSense App each morning for processing – collectively “OvuSense” (viO HealthTech Limited), having been offered the option at the start of their consultations. Each volunteer participated in the study for one embryo transfer cycle, and was recruited in one of three participating clinics: IVIRMA Madrid, IVIRMA Mallorca and IVIRMA Málaga.

### Study type

The study is observational and longitudinal in nature and based on the acquisition of prospective data.

### Study period and context

Each participant provided detailed medical history at the start of the study by way of a questionnaire. Informed consent was obtained at the start of the questionnaire process with the option to discontinue participation prior to use of OvuSense by denying consent.

Each participant was assigned a unique study id, and used the OvuCore sensor for the entire time they are at rest every night of their embryo transfer cycle from the day after cessation of menstruation until 7 days after their pregnancy test.

Serum progesterone P4 levels were established by blood draw up to a maximum of five times in the embryo transfer cycle on the following days:

1. The day of endometrial evaluation (day 8–12).
2. The embryo transfer day.
3. After 5 days of supplemented progesterone administration.
4. The day of the bHCG pregnancy test.
5. 7 days after the bHCG pregnancy test.

To optimize the convenience for the patient, the blood draw time for each of these days was from 14:00 (onwards) on the same day. Blood samples were obtained through venipuncture using a syringe according to standard clinical practice. Serum samples were tested with an electrochemiluminescence immunoassay (Cobas^®^ e 411 analyser, Roche diagnostics GmbH, Germany) in each participating clinic.

All patients in the study received micronized progesterone 400 (Cyclogest^®^, L.D. Collins & Co. Ltd.) supplemented twice daily in equal does of 400 mg, once in the morning, once at night. Estrogen therapy with oral dose Estradiol valerate (Estradiol Meriestra^®^, Novartis AG (Switzerland)) was 6 mg per day.

Serum samples were immediately analyzed after their collection. Remaining serum samples after the analysis were stored frozen at each participating IVIRMA clinic until the end of the study in case it is necessary to repeat the hormonal determination. At the end of this research study, the frozen blood samples will be destroyed. Blood sample data will be stored as electronic data in SIVIS (electronic clinical data system located at IVIRMA headquarters), following usual clinical practice.

### Reference population

The reference population was all women undergoing an embryo transfer with hormone replacement therapy for a frozen embryo transfer (FET) or egg donation cycles.

### Inclusion criteria

- Willing to enroll and use the OvuCore vaginal sensor on a nightly basis throughout one embryo transfer cycle as described in Study period and context.
- Actively committed to participating in IVF treatment.
- Currently using no form of contraception.
- Patients that need subcutaneous progesterone administration due to low levels of progesterone the embryo transfer day will also be included.

### Exclusion criteria

- Women with a history of recurrent vaginal irritation, vaginal infection or vaginismus (note: all class II regulatory required biocompatibility and cyto-toxicity tests have been completed on OvuCore).
- Previous diagnosis of adenomyosis.
- Previous history myomas.
- BMI > 30.
- Patients with previous low progesterone levels that need subcutaneous progesterone intake since the beginning of the progesterone administration.

### Intervention and follow-up

There was no intervention aiming to change the routine practice. Participants were advised to cease use of the vaginal sensor should irritation occur, or should they wish. In the case of cessation of use of OvuSense for whatever reason, the data to that point were retained in the study.

### Sample identification protocol

Results were not blinded but were recorded pseudonymized in an external database for further analysis. Once the blood sample was extracted, a code was assigned which was the only identification. The samples were pseudonymized, guaranteeing at all times the maintenance of the confidentiality of the data in accordance with the provisions of the GDPR.

### Main outcome measures

Raw Temperature = Body temperature in ºC, being the most representative temperature recorded for an overnight set of temperatures as determined by the OvuSense algorithm.

Smooth Temperature = Body temperature in ºC, being the moving mean Raw Temperature as determined by the OvuSense algorithm.

P4 (on each sampling day) = P4 blood serum progesterone result measured in ng/ml.

Positive or negative blood bHCG test.

Ongoing Pregnancy, Miscarriage or biochemical pregnancy.

OvuSense cycle flags: “Crash After Ovulation” or all other flags treated as “Normal”.

### Statistical methodology

An initial inspection of each individual’s temperature chart, with P4 values superimposed, and then a mean temperature chart with mean P4 values superimposed was made to inform further investigation of the data. A regression analysis was then carried out using ST as a predictor of P4 values, within a multilevel model, to account for between-patient variability. The specialist multilevel statistical software package MLwiN 3.10 (Charlton et al. 2024) [13] was used. ‘Agreement’ between ST on the day of blood sampling and P4 levels were then assessed using a Bland Altman plot (Bland & Altman 1986) [14]. The original multilevel regression model was then further refined to assess cross-correlation between ST and P4 and to investigate their relationship at the level of the individual participant. A further inspection of mean ST and mean P4 was charted for cycles flagged as ‘normal’ by OvuSense compared with cycles flagged as atypical by means of the ‘Crash After Ovulation’ flag. A final inspection of mean ST and mean P4 was charted for cycles with three outcomes: ‘Ongoing Pregnancy’ by the end of the embryo transfer cycle, ‘Negative bHCG Pregnancy Test’, and ‘Miscarriage or biochemical pregnancy’.

## Results

### Included results

Eighteen of 26 participants provided a full temperature curve for their embryo implantation cycle using OvuSense with sufficient P4 results to allow inclusion in the analysis. Of which, 12 participants had four to five P4 results (the fifth P4 normally only tested if a positive bHCG pregnancy test had occurred at the time of the fourth P4 test), three participants had three P4 results, and a further three participants had two P4 results. The effect of centre was ignored, and patients were treated as if from a single centre.

### Excluded results

Results from a further 8 participants were excluded from the analyses. Of those, one participant provided a full OvuSense temperature curve but no blood results were recorded, three participants left the study due to concerns over the vaginal OvuSense sensor, and four participants cancelled or abandoned their embryo implantation cycle.

### Initial analysis – inspection of individual charts

**Fig. 1:**
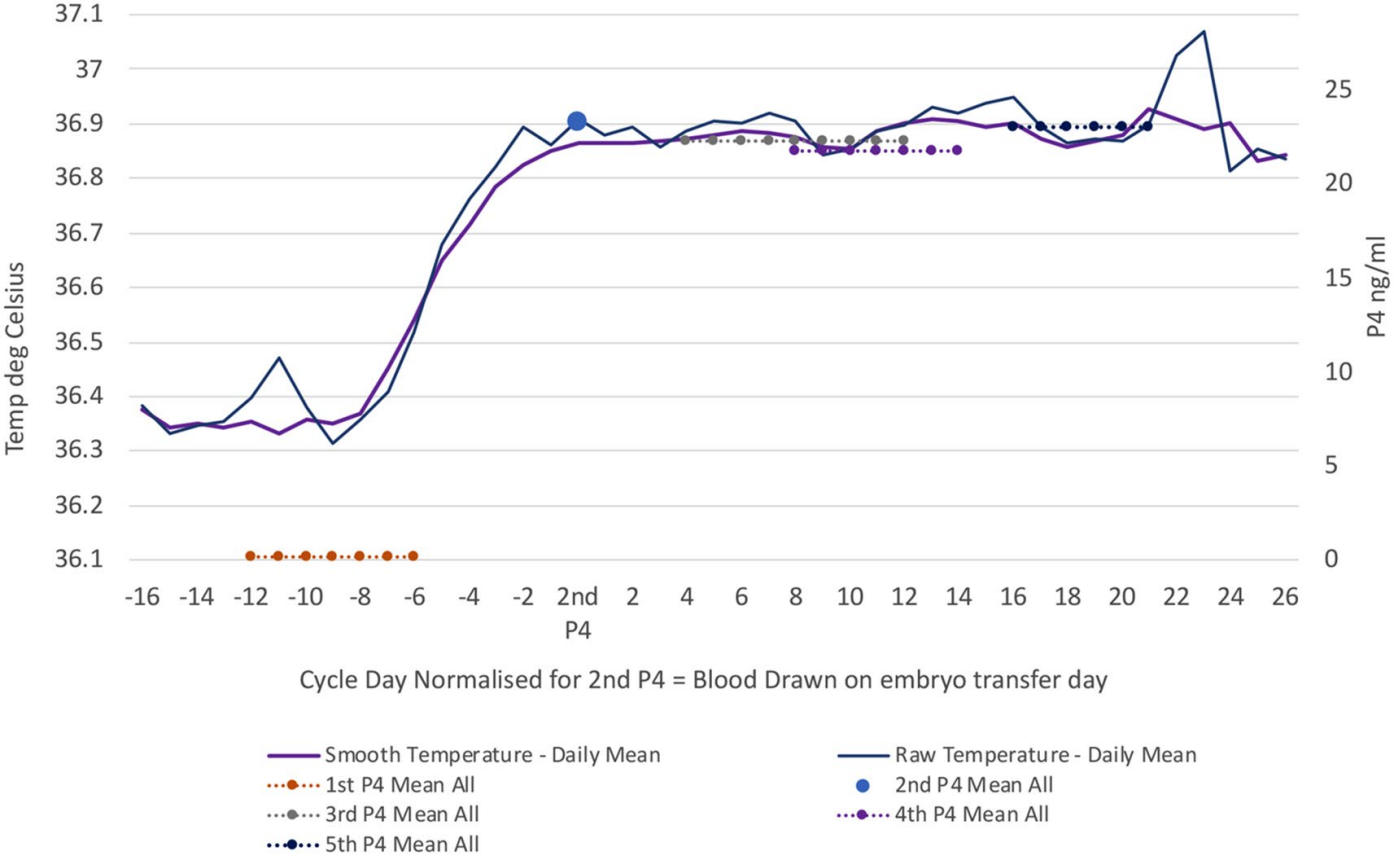
Daily mean OvuSense Raw and Smooth Temperature, registered to the 2nd P4 blood draw day, graphed against mean P4 value for each blood draw in ng/ml

For each of the 18 participants a time-based temperature chart was plotted, showing both the raw and ST, upon which the individual’s P4 measurements were superimposed. These charts were visually assessed for the relationship between temperatures and spot P4 measurements. Figure 1 shows the mean of the 18 charts, registered to the day of embryo transfer on the x-axis. The mean of the P4 values at each sampling time are shown superimposed and their spread shows the range of the period over which samples were taken for that sampling point. The temperature curves show the rapid rise associated with HCG trigger prior to embryo transfer, and as expected these reflect the rise from the P4 control values to the P4 values for the blood draw on day of embryo transfer. The luteal phase in these mean values shows a steady level of temperature reflecting very similar P4 values at each draw throughout the luteal phase.

### Simple regression and bland altman plot

Given the apparent relationship between temperature and P4 seen in Fig. 1, a simple regression analysis was performed with ST as the independent variable and LnP4 as the dependent variable. A natural log transform is a common requirement for hormone measurements to ensure that the assumptions required for the analysis are met. A regression within a multilevel model (MLwiN 3.10) was employed to account for the lack of independence of the repeated measurements made on the participants. The residuals from the regression were visually inspected and found to fit with the assumptions required for the model. The regression indicated a highly significant linear relationship between ST and LnP4 (Z = 14.738, *p* < 0.0001).

**Fig. 2:**
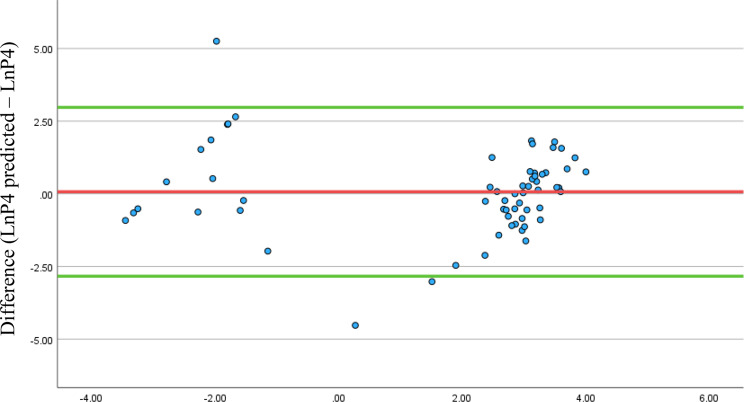
Bland Altman plot showing the agreement between the levels of LnP4 predicted from body temperature measured the day before blood sampling and LnP4 measured in the blood. The red line shows the mean of the differences (0.070) and the green lines the 95% confidence interval for the agreement between the measures

However, this was not unexpected given the clustering of high and low temperature and P4 values, so a Bland Altman plot was then constructed to assess the actual agreement between the measures. The Bland Altman plot in Fig. 2 shows a lack of agreement between the measures in aggregate.

A Bland Altman plot is appropriate if, for example, a single value of P4 is to be estimated using body temperature. However, in most real use cases daily body temperature measurements would be taken from one woman to assess *changes* in levels of P4, and in terms of regression, the multilevel model showed much of the residual variation was due to differences between participants. The multilevel model was therefore further developed as a “participant level model” to investigate this.

### Participant level model

Regression lines were fitted for each of the individual participants as the original simple regression had indicated that much of the residual variance was due to variability between participants (48%).

As before, LnP4 values were used but, on this occasion, the ST from the day before blood sampling was modelled as it showed a stronger relationship than temperature on the actual day of blood sampling (Chi-sq = 5.979, *p* = 0.015). Two data points had to be dropped from the analysis as they could not be matched to a ST from the day before sampling.

**Fig. 3:**
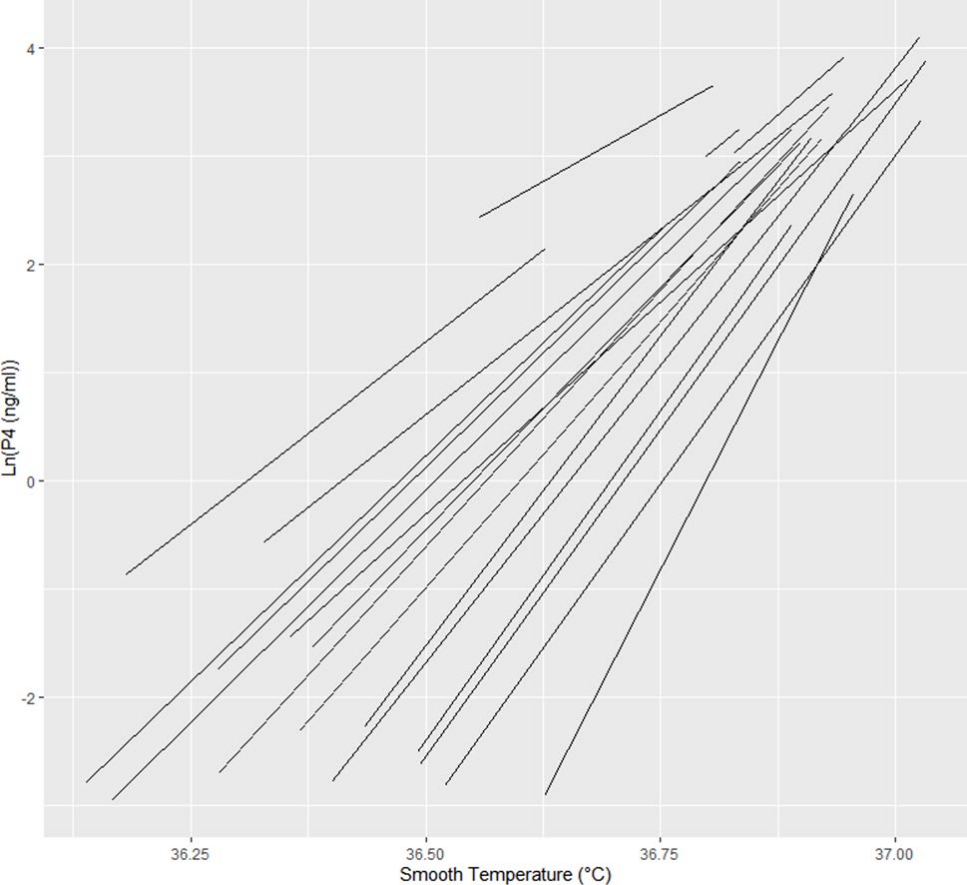
The estimated regression lines for each of the 18 individual participants, showing that the relationship is unique for each participant, but predictable once the starting temperature is known

The best fit model is shown in Fig. 3. As measured by the change in -2log-likelihood, the model in Fig. 3 was an improvement over the single slope model (Chisq = 9.024, df = 2, *p* = 0.011). There is a negative covariance (-3.098, SE 1.339) between individuals’ intercepts and slopes. This means that participants who started with higher progesterone levels (higher intercept) tended to have a slower increase in progesterone with temperature (shallower slope). This negative covariance corresponds to a negative correlation (-0.88) between intercept and slope, causing the individual progesterone-temperature relationship lines to converge at higher temperatures.

Thus, individual participants tended to have a unique relationship between temperature and P4. However, once the temperature for a given woman is being tracked, her offset is known and the tracking becomes more accurate to identify *changes* in LnP4 due to change of ST.

In order to further understand the importance of the starting temperature, and the relationship between temperature and progesterone through the cycle as a tool in assessing embryo transfer cycles, the temperature curves were then examined in respect of the cycle flags provided by OvuSense, and in terms of cycle outcomes.

### Graphical analysis for OvuSense cycle flags

**Fig. 4:**
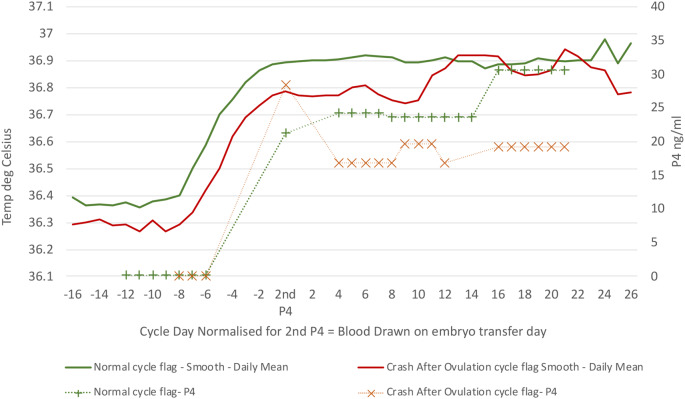
Daily mean OvuSense Smooth Temperature, registered to the 2nd P4 blood draw day for cycles flagged as “Normal” by OvuSense (denoted by the green curve) graphed against mean P4 value for each blood draw for those cycles flagged as “Normal” by OvuSense in ng/ml (denoted by the green dotted line with plus symbols); and daily mean OvuSense Smooth Temperature, registered to the 2nd P4 blood draw day for cycles flagged as atypical with the “Crash After Ovulation” flag by OvuSense (denoted by the red curve) graphed against mean P4 value for each blood draw for those cycles flagged as “Crash After Ovulation” by OvuSense in ng/ml (denoted by the red dotted line with ‘x’ symbols). Note: the span of the secondary y-axis for P4 is 0–40 ng/ml to accommodate the highest mean P4 value, and therefore different from the P4 span shown in Fig. 1 of 0–25 ng/ml

In addition to charting Raw and Smooth Temperature, OvuSense provides a number of flags indicating the type of temperature pattern shown in a cycle. The flag of particular interest for assessing the luteal phase is “Crash After Ovulation” [15]. The Crash After Ovulation flag is displayed by OvuSense when the final OvuSense Raw Temperature is more than 0.20 degrees Celsius (0.36 degrees Fahrenheit lower than the post ovulatory peak ST and has been shown to have a high odds ratio for risk of miscarriage [16]. To determine whether there was a relationship between ST values and P4 for the Crash After Ovulation flag, a similar analysis to Fig. 1 was produced. This graphed the results for all cycles flagged as Crash After Ovulation (five participants) for daily mean ST and mean P4 for each blood draw alongside daily mean ST and mean P4 for each blood draw for all remaining cycles which were deemed to be “Normal” (remaining 13 participants). This analysis is shown in Fig. 4.

The graphical analysis in Fig. 4. shows four main characteristics associated with cycles flagged by OvuSense with the “Crash After Ovulation” flag:

- Delayed rise associated with the HCG trigger shot.
- Higher P4 value at embryo transfer day.
- Lower P4 values associated with lower overall ST in the luteal phase after embryo transfer.
- Increase in ST later in the luteal phase not matched by increased P4 values.

### Graphical analysis for outcomes

In order to determine whether there was a relationship between OvuSense curves and outcome, a similar analysis to Fig. 4 was produced.

**Fig. 5:**
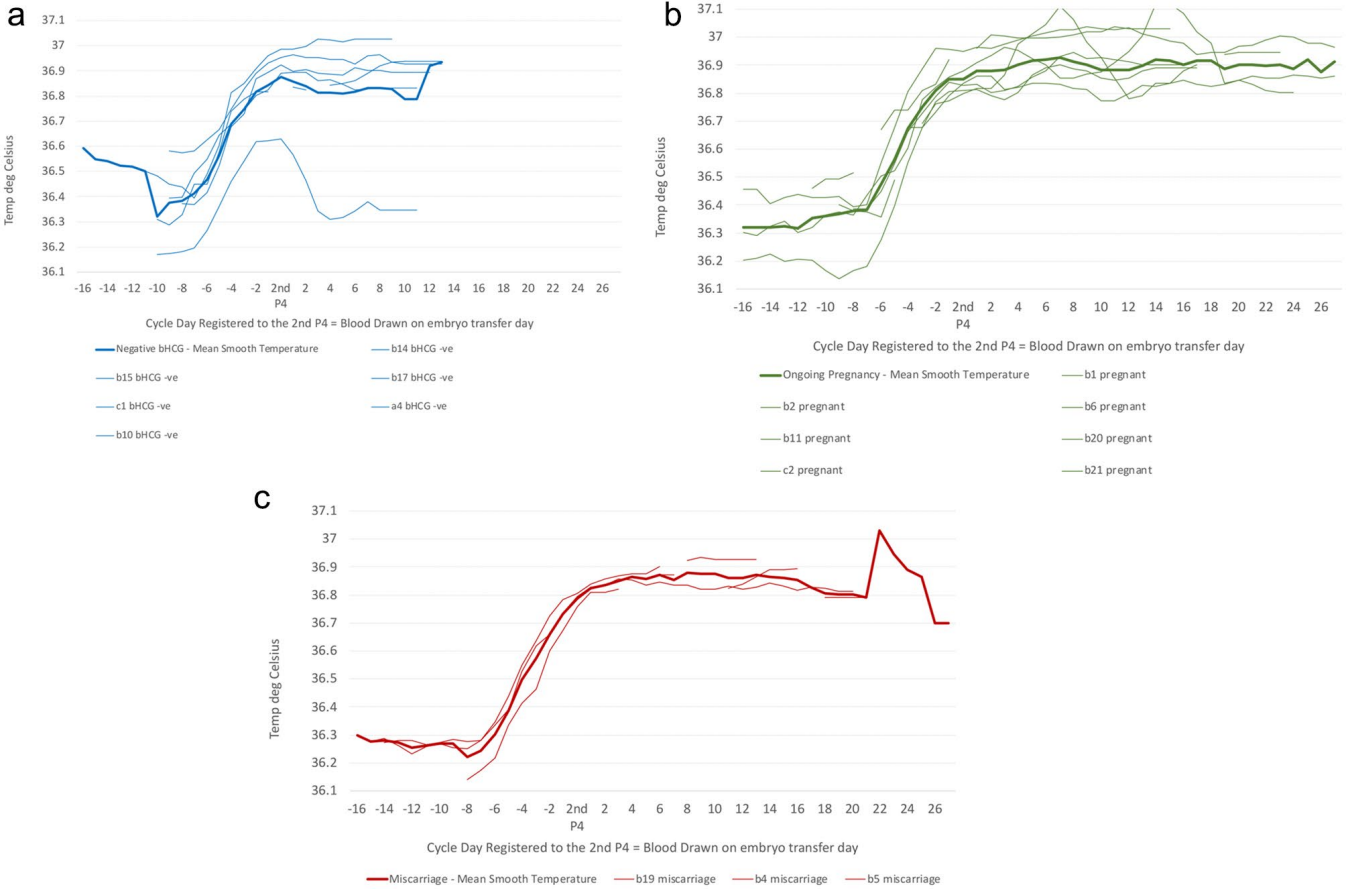
**a**. Daily OvuSense Smooth Temperature for each participant denoted by participant id with outcome of negative bHCG pregnancy test (thin blue curves), and mean daily OvuSense Smooth Temperature for those six participants (thick blue curve) registered to the 2nd P4 blood draw day. **b**. Daily OvuSense Smooth Temperature for each participant denoted by participant id with outcome of Ongoing Pregnancy at end of embryo transfer cycle (thin green curves), and mean daily OvuSense Smooth Temperature for those eight participants (thick green curve) registered to the 2nd P4 blood draw day. **C**. Daily OvuSense Smooth Temperature for each participant with outcome of Miscarriage or biochemical pregnancy, as entered in the participant notes by the clinic (thin red curves), and mean daily OvuSense Smooth Temperature for those three participants (thick red curve) registered to the 2nd P4 blood draw day

Firstly, the temperature patterns associated with outcomes were established. Figure 5a shows the results for all cycles for each patient with a negative bHCG pregnancy test (six participants) for daily ST for each patient and mean ST. Figure 5b shows the results for all cycles for each patient with Ongoing Pregnancy outcome at the end of the embryo transfer cycle (eight participants) for daily ST for each patient and mean ST Fig. 5c. shows the results for all cycles for each patient with a Miscarriage or biochemical pregnancy outcome at the end of the embryo transfer cycle (three participants) for daily ST for each patient and mean ST. One participant was excluded from this an analysis as the embryo transfer was aborted.

**Fig. 6:**
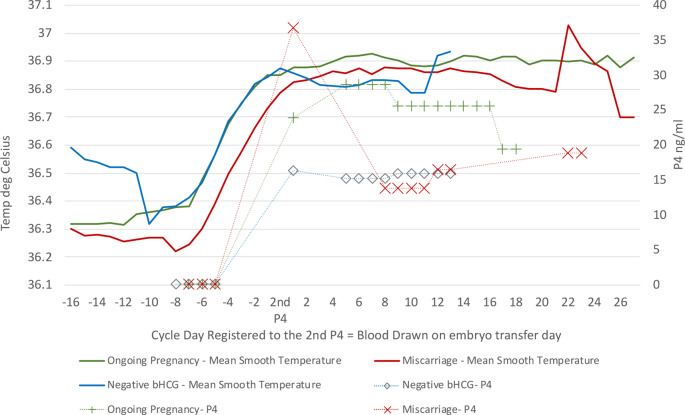
Daily mean OvuSense Smooth Temperature, registered to the 2nd P4 blood draw day for cycles with outcome of negative bHCG pregnancy test, as entered in the participant notes by the clinic (denoted by the blue curve) graphed against mean P4 value for each blood draw for those cycles in ng/ml (denoted by the dotted blue line with diamond symbols); daily mean OvuSense Smooth Temperature, registered to the 2nd P4 blood draw day for cycles with outcome of Ongoing Pregnancy, as entered in the participant notes by the clinic (denoted by the green curve) graphed against mean P4 value for each blood draw for those cycles in ng/ml (denoted by the green dotted line with plus symbols); and daily mean OvuSense Smooth Temperature, registered to the 2nd P4 blood draw day for cycles with outcome of Miscarriage or biochemical pregnancy, as entered in the participant notes by the clinic (denoted by the red curve) graphed against mean P4 value for each blood draw for those cycles in ng/ml (denoted by the dotted red line with ‘X’ symbols). Note: the span of the secondary y-axis for P4 is 0–40 ng/ml to accommodate the highest mean P4 value, and therefore the same as the span for Fig. 4, but different from the P4 span shown in Fig. 1 of 0–25 ng/ml

Secondly, these mean ST patterns were examined in comparison to the mean P4 results. Figure 6 shows the results for all cycles with a negative bHCG pregnancy test (six participants) for daily mean ST and mean P4 for each blood draw, alongside daily mean ST and mean P4 for each blood draw for cycles with an Ongoing Pregnancy outcome at the end of the embryo transfer cycle (eight participants), and daily mean ST and mean P4 for each blood draw for cycles with a Miscarriage or biochemical pregnancy outcome at the end of the embryo transfer cycle (three participants). The graphical analyses in Fig. 5. and 6. shows differences between the ST curves based on outcomes as follows:

- The individual patient patterns appear to follow a similar trend to the mean ST pattern for the negative bHCG cohort with one outlier with particularly low temperatures throughout the cycle, and a sharper drop in temperature after embryo transfer. There is a greater variability in individual patient patterns when compared with the mean ST pattern for the Ongoing Pregnancy cohort, though a similar trend. The individual patient patterns follow a very a similar trend to the mean ST pattern Miscarriage or biochemical pregnancy cohort, although with only three participants in the cohort.
- The Negative bHCG pregnancy test ST curve appears to match the pattern for Ongoing Pregnancy ST curve until embryo transfer day, after which there is a drop in ST which is also matched by lower P4 values.
- The Miscarriage or biochemical pregnancy ST curve looks similar to the “Crash After Ovulation” curve in Fig. 4, with a delay in ST rise of 2–3 days after HCG trigger and lower overall ST values through the luteal phase.
- As with the “Crash After Ovulation” ST curve in Fig. 4. this is matched with a higher P4 value at embryo transfer day, and overall lower luteal phase P4 values.

## Discussion

### Main findings

Continuous body temperature monitoring using OvuSense provides a minimally invasive, accurate method for predicting progesterone fluctuations. Graphical analysis suggests differences between ST curves associated with the cycle flags provided by OvuSense, and that there may be differences in ST curves based on outcomes.

This approach can potentially improve IVF cycle management by replacing some or all P4 blood tests, saving patients and clinics the inconvenience of having to draw blood on specified days during the cycle. Moreover, the information appears to be available to clinicians and patients one day in advance of when a blood sample would normally be taken.

### Interpretation

The study supports the concept of integrating digital biomarkers which can be used in the home and queried remotely by clinicians into clinical practice to enhance patient care, and has potentially wider implications for the replacement of progesterone P4 testing in the monitoring of women’s health and associated conditions.

#### Strengths and limitations

Study participants had a consistent treatment and measurement protocol, accounting for potential variability in a general population being tested for the relationship between P4 and body temperature through a single menstrual cycle.

However, this is also a limitation in that it seeks to only compare these women. The hormone therapy and supplementation for the study cohort may be expected to create a more consistent outcome in hormone measurement, and body temperature as a proxy. Nonetheless, there appears to be value in the results for this cohort.

## Conclusion

### Practical recommendations

OvuSense is a potentially valuable tool for monitoring progesterone levels by acting as an accurate proxy for P4 that can be reviewed by clinicians remotely. It offers potential to improve patient comfort, reduce inconvenience, and minimize errors by removing some or all of the P4 blood tests required during an IVF cycle.

#### Research recommendations

Progesterone is known to be a useful biomarker for predicting pregnancy outcomes, with varying suggested cutoffs: levels below 6 ng/ml having a high positive predictive value for the diagnosis of a failing pregnancy, over 19 ng/ml ‘strongly’ associated with a viable pregnancy [17], and a suggested cutoff for embryo transfer day in the case of FET cycles of 10 ng/ml [18]. Although higher optimal luteal phase serum P4 levels have also been suggested [19]. 

This study has established a strong linear relationship between LnP4 and ST. The participant level model analysis shows that the starting ST for levels of P4 varies among participants, but there is a consistency of slope for ST. The individual relationship between temperature and progesterone for each participant is not a surprise given the known variability between women for P4 itself [17–19].

The results would indicate that the differential in ST could be best used to determine changes in P4, rather than absolute values. The cutoff for P4 values indicating positive (Ongoing Pregnancy) vs. negative (negative pregnancy test or Miscarriage) outcomes in this study would appear to be around 20 ng/ml with a difference between the P4 values being around 10 ng/ml. Additional research is required to confirm the identifiable differences in ST curves related to negative outcomes, and the potential to indicate an alteration in embryo transfer protocol.

The authors do not believe that supplemented progesterone alters the relationship between ST and LnP4 *per se*, and that ST represents the physiological level of P4 whether naturally occurring or because of supplementation.

Within the context of IVF, similar associations between the ST and LnP4 in natural cycles (for endometrial preparation) might therefore be expected. Likewise, the patterns observed in Figs. 4, 5 and 6 might be found to occur consistently for each individually, raising the possibility of these patterns providing a potential prognostic indication of outcome. However, both these questions remain unanswered, and a further study would be required to establish this.

Equally, a broader assessment of continuous body temperature measurement compared with P4 throughout the menstrual cycle is recommended. P4 measurement in the general population is impractical, often involves a single measurement during a cycle, and can fail to provide useful understanding of cycle progress, such as determining if a patient is ovulatory if blood is drawn on the wrong day of the cycle [20].

## Data Availability

The datasets generated and analysed during the current study are not publicly available as each individual patient outcome has been logged which might compromise data privacy under GDPR legislation, but are available from the corresponding author with outcome information removed on reasonable request.
